# An Analysis of Mass Screening Strategies Using a Mathematical Model: Comparison of Breast Cancer Screening in Japan and the United States

**DOI:** 10.2188/jea.JE20140047

**Published:** 2015-02-05

**Authors:** Miwako Tsunematsu, Masayuki Kakehashi

**Affiliations:** Department of Health Informatics, Graduate School of Biomedical and Health Sciences Hiroshima University, Hiroshima, Japan; 広島大学大学院 医歯薬保健学研究院 健康情報学研究室

**Keywords:** breast cancer, mass screening, mathematical model, benefit, harm

## Abstract

**Background:**

Although the United States Preventive Services Task Force (USPSTF) downgraded their recommendation for breast cancer screening for women aged 40–49 years in 2009, Japanese women in their 40s have been encouraged to attend breast cancer screenings since 2004. The aim of this study is to examine whether these different mass-screening strategies are justifiable by the different situations of these countries and to provide evidence for suitable judgment.

**Methods:**

Performance of screening strategies (annual/biennial intervals; initiating/terminating ages) was evaluated using a mathematical model based on the natural history of breast cancer and the transition between its stages. Benefits (reduced number of deaths and extended average life expectancy) and harm (false-positives) associated with these strategies were calculated.

**Results:**

Additional average life expectancy by including women in their 40s as participants were 13 days (26%) and 25 days (22%) in Japan and the United States, respectively, under the biennial screening condition; however, the respective increases in numbers of false-positive cases were 65% and 53% in Japan and the United States. Moreover, the number of screenings needed to detect one diagnosis or to avert one death was smaller when participants were limited to women of age 50 or over than when women in their 40s were included. The validity of including women in their 40s in Japan could not be determined without specifying the weight of harms compared to benefits.

**Conclusions:**

Whether screening of women in their 40s in Japan is justifiable must be carefully determined based the quantitative balance of benefits and harms.

## Abstract (ja)

**【背景】:**

2009年、米国予防医学専門委員会（USPSTF）が40歳代の定期的なマンモグラフィ検診に関して推奨グレードを下げたが、わが国では、2004年から40歳代の乳がん検診の受診が推奨されている。本研究の目的は、両国の異なる背景を考慮すれば、最適な検診条件が異なるのかどうか、根拠を示して検討することである。

**【方法】:**

乳がん自然史に基づいて、いくつかの段階を時間の経過とともに確率的に移行するように設定された数理モデルを使用して、様々な条件下（毎年と隔年の検診間隔、検診の開始年齢と終了年齢）での検診成績を評価した。利益としては、減少した死亡者数と延長された平均余命、不利益としては、偽陽性者数を計算した。

**【結果】:**

隔年検診で、検診開始年齢を50歳代から40歳代にした場合、平均余命の延長効果は、日本では13日（26％）、米国では25日（22％）であった。一方、偽陽性者数は、日本では65％、米国では53％増加した。さらに、1人の乳がんを発見するために必要な検診受診者数と1人の乳がん死亡を防ぐために必要な受診者数は、40歳代を含めた時より50歳代以上に限定した方がより少なかった。わが国で40歳代を乳がん検診の対象年齢にするかどうかの正当性は、利益と不利益の評価を明確にしなければ決定できなかった。

**【結論】:**

わが国で40歳代を検診の対象年齢にするかどうかは、利益と不利益の量的なバランスにかかっており、慎重に決定することが必要である。

## INTRODUCTION

In 2009, the United States Preventive Services Task Force (USPSTF) downgraded the recommended level of breast cancer screening for women aged 40–49 years to grade C (a recommendation for selective screening based on professional judgment and patient preferences, based on at least moderate certainty of a small net benefit) because the benefits of screening mammography were equivalent between women aged 40–49 years and those aged 50–59 years, but false-positive results were much more common in women aged 40–49 years.^1^^–^^3^ This evoked multi-disciplinary controversy among those concerned with breast cancer screening.^4^^–^^6^ It was pointed out that the attitudes of authors who opposed the guideline were related to their specialty.^7^ In contrast, breast cancer screening is currently recommended for women aged 40–49 years in Japan. The Japan Association of Breast Cancer Screening recognizes that the change in the USPSTF’s recommendations is evidence-based and is fairly appropriate but has manifested its view that the update is based on data applicable to the United States and not directly applicable to Japan.^8^ Guidelines proposed so far have supported the invitation of women in their 40s.^9^^–^^11^

Incidence and mortality of breast cancer in Japan and the United States differ: incidence peaks at ages 45–49 years and mortality peaks at ages 55–64 years in Japan, whereas peak breast cancer incidence and mortality rates in the United States occur in women aged 60 years or older.^12^ Thus, we cannot simply apply results from the United States to Japan, and differences in the epidemiology of breast cancer between both countries should be investigated.

In the United States, the Cancer Intervention and Surveillance Modeling Network (CISNET), supported by the National Cancer Institute, has examined effects of modeling countermeasures in breast cancer screening.^13^^,^^14^ The updated guideline recognizes the results from mathematical modeling research conducted by CISNET as important evidence, in addition to common evidence reports.^3^ The usefulness of such modeling is also recognized in Japan, where Ohnuki, Iinuma, and other researchers have investigated the effects of cancer screening using mathematical models and have contributed to the determination of screening strategies.^15^^–^^18^ However, little research has quantitatively assessed the benefits and the harmful effects of breast cancer screening simultaneously. In addition, no calculation is available of the extended average life expectancy when excluding breast cancer from all causes of death, although the extended average life expectancy is 3.03 years when excluding malignant neoplasms from all causes of death in Japan.^19^ Therefore, the establishment of an optimal breast cancer screening strategy specifically for Japanese women requires research using a mathematical model to quantitatively assess the effects of screening.^20^

The present study aims to assess quantitative benefits (reduced number of deaths and extended average life expectancy) and harms (false-positive results) of breast cancer screening to provide evidence for suitable judgment.

## METHODS

### Mathematical model of mass screening

**Figure 1.**
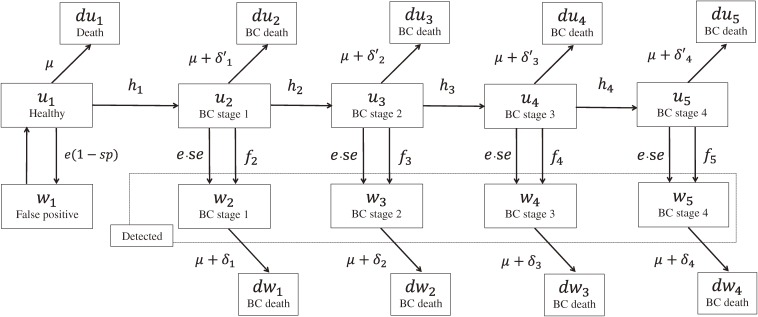
A mathematical model of breast cancer screening consisting of 12-month cycles of 10 health states that simulate the theoretical natural history of breast cancer, comprising the following seven structures: *u*_1_: healthy; *w*_1_: false positive; *u*_2_–*u*_5_: undetected breast cancer (stages 1–4); *w*_2_–*w*_5_: detected for breast cancer (stages 1–4) through screening or outpatient care; *du*_1_: died from a cause other than breast cancer; *du*_2_–*du*_5_^a^: undetected and died of breast cancer; *dw*_1_–*dw*_4_^a^: detected and died of breast cancer. Stage classifications used here are those published by the Union for International Cancer Control (UICC) for Japanese data and by the American Joint Committee on Cancer (AJCC) for United States data. ^a^Death from causes other than breast cancer (*μ*) is excluded. BC, breast cancer.

Analyses were carried out using a mathematical model of mass screening and were performed with Mathematica 8.0 computational software (Wolfram Research, Champaign, IL, USA). The basic structure of the model is shown in Figure 1. In this model, based on the natural history of breast cancer, the number of women of each age specified by stage of breast cancer was calculated year by year. The number of women with breast cancer was also specified by treatment status (detected or undetected). Women with detected breast cancer were considered to be on treatment, while women with undetected breast cancer were deemed not on treatment. Women become one year older each year and subsequently may develop breast cancer. The transition rate from one stage to the next is summarized in Table 1.^21^^–^^36^ Rates at which women die of breast cancer or from other causes according to mortality rates were obtained from Vital Statistics.^26^^–^^29^ Incidence rates were also obtained from population-based cancer registries.^21^^,^^22^ Incidence rates of breasts cancer in women of each age were calculated by the conversion of data from a 5-year age classification.

**Table 1.** Parameters used in the screening model

Parameters			Values used in the model		Data source

			Japan	United States	
Trassition probabilities					
	Progression of undetected breast cancer^a^				
	*h*_1_	Healthy to stage 1 (incidence rate: age-specific, per 100 000)	0.0–0.001 46	0.0–0.004 21	21, 22
	*h*_2_	Stage1 to stage2	0.22	0.43	
	*h*_3_	Stage2 to stage3	0.06	0.12	
	*h*_4_	Stage3 to stage4	0.01	0.05	
	Transition rate of women with undetected breast cancer to undergo outpatient care^a^				23–25
	*f*_2_	Stage 1 to outpatient care	0.07	0.04	
	*f*_3_	Stage 2 to outpatient care	0.12	0.05	
	*f*_4_	Stage 3 to outpatient care	0.11	0.09	
	*f*_5_	Stage 4 to outpatient care	0.40	0.16	
	Stage-specific mortality rate of women with detected breast cancer^b^				26, 27
	*δ*_1_	Stage1	0.008	0.012	
	*δ*_2_	Stage2	0.021	0.043	
	*δ*_3_	Stage3	0.062	0.107	
	*δ*_4_	Stage4	0.230	0.273	
	Stage-specific mortality rate of women with undetected breast cancer (*δ'* = 1.5*δ*)^c^				
	*δ*′_1_	Stage1	0.011	0.019	
	*δ*′_2_	Stage2	0.032	0.064	
	*δ*′_3_	Stage3	0.093	0.161	
	*δ*′_4_	Stage4	0.344	0.409	
	Mortality rate of other causes				
	*μ*	Mortality rate (age-specific, per100 000)	0.0001–0.3136	0.0001–0.2948	28, 29

Stage distribution of breast cancer					
	Outpatient care, %				
		Stage1	41.8	26.3	
		Stage2	46.3	38.8	
		Stage3	9.2	23.0	
		Stage4	2.7	11.9	
	Screening, %				23–25
		Stage1	69.9	63.8	
		Stage2	27.3	29.8	
		Stage3	2.3	5.1	
		Stage4	0.6	1.3	

Screening variables					
	*e*	Screening rate, %	0, 30, 100	0, 50, 100	30, 31, Assumed
	*se*	Screening sensitivity, %	81.5	83.5	32, 33
	*sp*	Screening specificity by age, %	90.4–94.7	90.2–93.1	2, 34
	*sd*	Screening detection rate, %	0.32	0.47	35, 36
	*scmin*	Screening initiating age	Ages 40, 45, 50, 55, and 60		Assumed
	*scmax*	Screening terminating age	Ages 69, 74, 79, and 84		Assumed
	*interval*	Screening interval	Annual and biennial		Assumed

^a^Estimated from breast cancer stage distribution.

^b^Estimated from survival rate by stage of breast cancer.

^c^Mortality rate of women with undetected breast cancer set to from 1.0 to 3.0 times the mortality rate of women with detected breast cancer for sensitivity analysis.

The present model has the following assumptions: At the beginning of the simulation, a population of 100 000 women is in a healthy state (*u*_1_). Breast cancer progresses over time when a shift is made from a healthy state (*u*_1_) to breast cancer (*u*_2_). Women who were detected to have breast cancer through screening or outpatient care (*w*_2_–*w*_5_) do not move to another stage nor return to being untreated. Women with breast cancer (*w*_2_–*w*_5_) are categorized as the screening detection group and the outpatient care detection group, according to detection history. However, this categorization is made separately for each age group. Breast cancer shifts sample members from a healthy state (*u*_1_) to onset (*u*_2_) in accordance with age-specific incidence rates of breast cancer (*h*_1_). However, effects of age are not considered for transition rates (*h*_2_–*h*_4_) in the subsequent progression of breast cancer. Women treated for breast cancer (*w*_2_–*w*_5_) die at the mortality rate (*δ*), which is assumed to be greater (*δ*′ = 1.0–3.0*δ*) in untreated women (*u*_2_–*u*_5_) than in women who received treatment (*w*_2_–*w*_5_).

### Parameters estimation

Transition rates (*h*_2_–*h*_4_, *f*_2_–*f*_5_) in undetected women (*u*_2_–*u*_5_) were estimated based on the stage distributions of women whose breast cancer was detected by outpatient care and screening according to the maximum likelihood method. The sources of stage distributions were data published by the Japanese Breast Cancer Society^23^ in Japan and the Breast Cancer Surveillance Consortium (BCSC)^24^ and the National Cancer Data Base (NCDB)^25^ in the United States.

The mortality rates (*δ*) per year for patients with breast cancer were calculated based on 10-year survival rates from published sources.^26^^,^^27^ Survival rates were logarithmically converted; mortality rates for breast cancer per year at each stage were estimated using the least-square method.

### Validity of the estimated parameters

Regarding the breast cancer screening rate (*e*), data from the 2007 Comprehensive Survey of Living Conditions^30^ were used for Japan, while those from the Breast Cancer Facts & Figures 2011–2012, published by the American Cancer Society,^31^ were used for the United States. Screening rates at ages 40 to 65 years were set to be approximately 30% (per year) for Japan and approximately 50% (per year) for the United States, as actually observed in each country. Screening rates after age 65 were adjusted to gradually decrease in tandem with aging. After the estimation of parameters, predictions obtained by the model were compared with statistically reported data of incidence,^21^^,^^22^ mortality,^37^^,^^38^ and stage distribution of breast cancer.^23^^–^^25^ In checking validity, only the sum of cases of detected breast cancer (*w*_2_–*w*_5_) was compared with the statistically reported number of cases, while both detected (*w*_2_–*w*_5_) and undetected (*u*_2_–*u*_5_) breast cancer cases were used to compare screening strategy performances.

### Evaluation of the performance of mass screening strategies

The performance of mass screening strategies was evaluated in terms of both benefits and harms. Benefits were calculated as reduced number of deaths and extended average life expectancy attributable to screening. Average life expectancy at age *x* was calculated from total number of person-years lived after age *x* divided by the number of individuals alive at age *x*. A half year was added because individuals who died at any age lived more than a half year on average. The theoretical maximal benefit of breast cancer screening was calculated as the difference between average life expectancy without breast cancer death and that without screening. The harms of breast cancer screening are represented mainly by false-positive results and overdiagnosis. In this study, we considered primarily false-positive results by age. We also calculated the number of screenings needed to detect one diagnosis or to avert one death.

### Comparison of annual vs. biennial screening and the range of ages for screening

To examine the effect of the range of ages screened, absolute benefit (reduction in the number of deaths and days of extended average life expectancy) and relative benefit (proportion of death reduction and extended average life expectancy) were compared between 100% screened and unscreened women under different screening strategies. To examine the effect of screening intervals, the ratio of biennial-to-annual benefit were calculated (with the proportion of death reduction maintained). Furthermore, to examine relative benefit, regression analysis was performed using ages to initiate and terminate screening as independent variables and benefits as dependent variables. The number of women with false-positive results and the number needed to screen to detect one diagnosis or to avert one death were estimated to examine the harm of screening strategies. Finally, to compare the benefits to the population and patients with breast cancer, the patients’ average life expectancy was calculated when screened and when unscreened. Statistical analyses were performed with PASW Statistics 18 (SPSS Japan Inc., Tokyo, Japan). *P* < 0.05 was considered statistically significant.

### Sensitivity analysis

To assess the uncertainties surrounding key variables in the model, a sensitivity analysis on the mortality rate of women with undetected breast cancer was performed. The variable was tested in univariate sensitivity analyses by mortality rate of women with undetected breast cancer (*δ*′) from 1.0 to 3.0 times of the mortality rate of women with detected breast cancer (*δ*).

## RESULTS

### Validity of the estimated parameters

**Figure 2.**
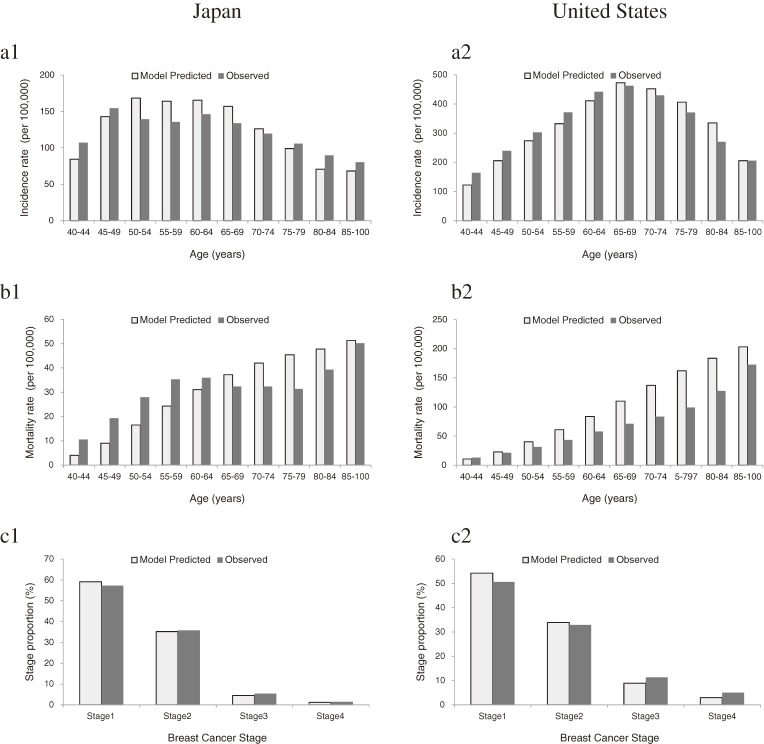
Model-predicted and observed statistics on age-specific incidence (a), mortality (b), and stage distribution of breast cancer (c) in Japan and the United States. A population of 100 000 women was traced from age 0 to 100 years. Observed statistics on incidence^a^ peaked (154.5 per 100 000 women) in those age aged 45–49 years in Japan, whereas the incidence increased continuously from age 45 and peaked (433.1 per 100 000 women) in those aged 75–79 years in the United States. Observed statistics on mortality tended to increase with age in both Japan and the United States. Differences in mortality between Japan and the United States were marked in women aged 50 years or older. ^a^Excluding carcinoma in situ.

The total number of patients with breast cancer in Japan as predicted with this model was 6091. The incidence rates of breast cancer in Japan as calculated using the statistically reported incidence and as predicted with this model were 69.9 and 71.0 patients per 100 000 women, respectively (Figure 2-a1). The total number of breast cancer deaths in Japan as predicted with the model was 1425. The mortality rates of breast cancer as calculated using mortality statistics and as predicted with this model were 17.6 and 16.6 deaths per 100 000 women, respectively (Figure 2-b1). Therefore, this model seemed to have successfully predicted observed statistics. In contrast, the total number of patients with breast cancer in the United States as predicted with this model was 13 417. The incidence rates of breast in the Unites States cancer as calculated using incidence statistics and as predicted with this model were 165.4 and 167.8 patients per 100 000 women, respectively (Figure 2-a2). The total number of breast cancer deaths in the United States as predicted with this model was 3723. The breast cancer mortality rates as reported using mortality statistics and as predicted with this model were 35.3 and 46.6 deaths per 100 000 women, respectively (Figure 2-b2). Therefore, the model of this study seems to be less successful in a United States population, although the numerical gap between predicted and observed morality rates was relatively small for both countries. The distribution of disease stage as predicted with this model nearly coincided with that reported by observed statistics in both countries (Figures 2-c1, c2).

### Extended life expectancy and theoretically maximal effect of breast cancer screening

If no women died of breast cancer, average life expectancy would be 88.15 years and 83.03 years in Japan and the United States, respectively. In the population without breast cancer screening (60-year follow-up from age 40), the total number of breast cancer deaths and average life expectancy in Japan were 2657 and 87.72 years, respectively. The total number of breast cancer deaths and average life expectancy in the United States were 9238 and 81.91 years, respectively. The theoretical maximum benefits of screening in Japan and the United States were 157 days (88.15 − 87.72 = 0.43 years) and 408 days (83.03 − 81.91 = 1.12 years), respectively. In addition, the extended average life expectancy was calculated among only patients with breast cancer. Average life expectancy when the patients with breast cancer were screened in the group aged 40–74 years under annual screening conditions was compared with that when they were unscreened, in both Japan and the United States. Average life expectancy of patients with breast cancer increased by 8.6 years and 12.6 years, respectively.

### Comparison of annual vs. biennial screening

Benefits were compared with harms when screened (assuming 100% compliance) under different screening conditions and when unscreened in *δ*′ = 1.5*δ* (Table 2). Changes in death reduction between annual and biennial screening intervals were compared. The biennial screening strategy maintained 82% (range: 80%–84%) and 76% (range: 74%–79%) of the death reduction obtained by annual screening in Japan and the United States, respectively, while reducing the ratio of false-positive to true-positive mammography results to about half (0.50–0.52 in both Japan and the United States) of that seen under an annual screening schedule.

**Table 2.** Benefits and harms of breast cancer screening by different ages for initiating and terminating screening

Strategy	Screenings, women^a^	Benefit					Harm		

		Number of deaths^a^	Reduced number of deaths	Death reduction, %	Maintained death reduction, %^b^	Extended average life expectancy, day^c^	False-positive results^a^	Number need to screen to detect one diagnosis	Number need to screen to avert one death
Japan									

Biennial screening									
No breast cancer	—	—	—	—	—	—	—	—	—
No screening	—	2657	—	—	—	—	—	—	—
40–69 y	1 443 763	1821	836	31	80	58	105 162	218	793
50–69 y	947 159	1952	705	27	81	45	57 470	149	485
40–74 y	1 704 723	1695	962	36	83	62	120 820	239	1006
50–74 y	1 208 118	1825	832	31	84	49	73 127	176	662
40–79 y	1 867 271	1636	1021	38	82	63	130 573	251	1141
50–79 y	1 370 666	1764	893	34	83	51	82 880	191	777
Annual screening									
40–69 y	2 877 439	1612	1045	39	—	72	209 656	407	1785
50–69 y	1 885 811	1787	870	33	—	55	114 430	278	1055
40–74 y	3 311 710	1491	1166	44	—	76	235 712	439	2221
50–74 y	2 320 081	1665	992	37	—	59	140 486	320	1393
40–79 y	3 717 061	1406	1251	47	—	78	260 033	466	2644
50–79 y	2 725 432	1582	1075	40	—	61	164 807	355	1723

United States									

Biennial screening									
No breast cancer	—	—	—	—	—	—	—	—	—
No screening	—	9238	—	—	—	—	—	—	—
40–69 y	1 401 446	6926	2312	25	74	137	123 917	119	202
50–69 y	906 670	7202	2036	22	74	112	75 422	80	126
40–74 y	1 627 584	6260	2978	32	78	157	139 521	118	260
50–74 y	1 132 808	6536	2702	29	79	132	91 025	85	173
40–79 y	1 757 771	5902	3336	36	76	164	148 504	117	298
50–79 y	1 262 995	6180	3058	33	77	139	100 008	87	204
Annual screening									
40–69 y	2 784 694	6098	3140	34	—	183	246 301	210	457
50–69 y	1 797 511	6496	2742	30	—	147	149 544	142	277
40–74 y	3 159 597	5403	3835	42	—	203	272 169	208	585
50–74 y	2 172 414	5801	3437	37	—	167	175 412	149	374
40–79 y	3 482 743	4856	4382	47	—	214	294 466	205	717
50–79 y	2 495 560	5253	3985	43	—	178	197 709	152	475

^a^Screenings, number of deaths, and false-positive results are all represented as the sum total (60-year follow-up from age 40). Death reduction and average life expectancy extension were compared between 100% screened and unscreened women. Mortality rate of women with undetected breast cancer is 1.5 times that of women with detected breast cancer. ^b^To examine the effect of screening intervals, the ratio of biennial-to-annual benefit were calculated (with the proportion of death reduction maintained). ^c^The theoretical maximal benefits of screening were 157 days (88.15 − 87.72 = 0.43 years) and 408 days (83.03 − 81.91 = 1.12 years) in Japan and the United States.

### Comparison of the range of ages for screening

Relative benefits of breast cancer screening at different ages to initiate and terminate screening are shown in Table 3. More benefit (proportion of death reduction) was obtained when initiating screening 1 year younger than when terminating screening 1 year older in Japan. In the United States, the opposite tendency was observed.

**Table 3.** Relative benefits of breast cancer screening at different ages for initiating and terminating screening

Screening strategy^a^		B-coefficient	β-coefficient	*P*-value	Adjusted *R*^2^
Death reduction, %^b^					
Japan	initiating ages^c^	−0.696	−0.771	0.000***	0.93
	terminating ages^d^	0.598	0.497	0.000***	
United States	initiating ages	−0.401	−0.473	0.000***	0.95
	terminating ages	0.900	0.797	0.000***	
Average life expectency extension, %^b^					
Japan	initiating ages	−1.078	−0.932	0.000***	0.93
	terminating ages	0.287	0.186	0.052	
United States	initiating ages	−0.829	−0.835	0.000***	0.93
	terminating ages	0.526	0.398	0.002**	

***P < 0.001, **P < 0.01, *P < 0.05.

^a^Screening interval is biennial.

^b^Proportion of death reduction and extended average life expectancy were compared between 100% screened and unscreened women.

^c^Ages to initiate screening were set at 40, 45, 50, or 55 to 60 years.

^d^Ages to terminate screening were set at 69, 74, or 79 to 84 years.

On evaluating screening benefits from the viewpoint of extended average life expectancy, the comparative results were markedly different. In both countries, it was more beneficial to initiate screening at a younger age than to terminate screening at an older age, with the benefit of initiating screening at a younger age slightly greater in Japan than in the United States.

### Efficiency of screening

In order to assess the efficiency of screening, we analyzed the number of screenings required to detect one diagnosis or to avert one death. It is notable that the number of screenings required to detect one diagnosis in Japan is almost twice that needed in the United States, because the incidence rate in Japan is smaller than that in the United States. As to the number of screenings required to avert one death, the gap expands to nearly four-fold, because the mortality rate in Japan is much lower than that in the United States. The number of screenings required to detect one diagnosis under biennial screening is smaller than that under annual screening, because new patients emerging over two years are detected on only one screening occasion. The number of screenings required to detect one diagnosis in the group aged 40–74 years (vs. 50–74 years) in *δ*′ = 1.5*δ* increased by 36% and 39% in Japan and the United States, respectively, under biennial screening conditions. The number of screenings needed to avert one death in the group aged 40–74 years (vs. 50–74 years) in *δ*′ = 1.5*δ* increased by 52% and 50% in Japan and the United States, respectively, under biennial screening conditions.

### Sensitivity analysis

**Figure 3.**
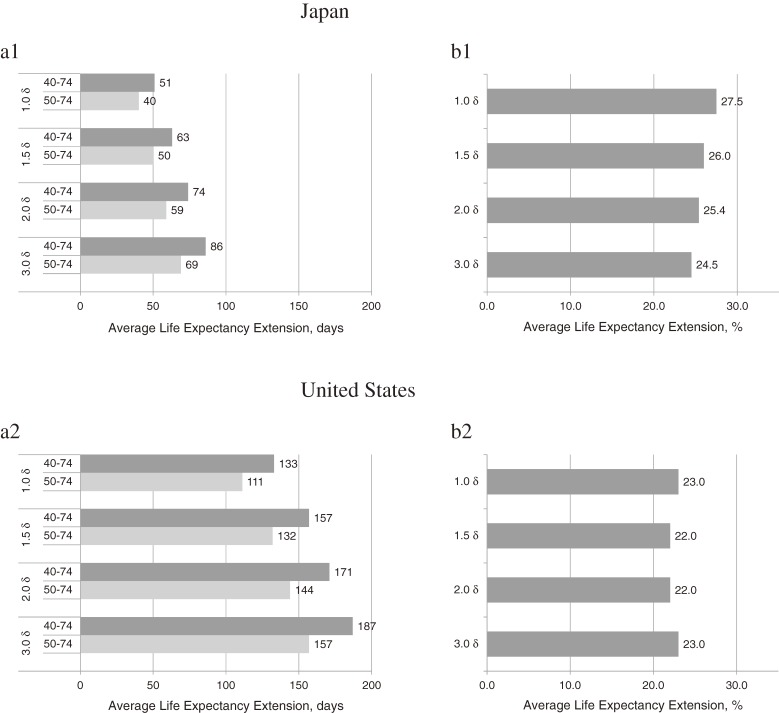
Average life expectancy extension in 40-to-74-year age group (vs. 50–74 years) in Japan and the United States; (a) absolute effect; (b) relative effect^a^. ^a^Relative effect of average life expectancy extension was calculated; in Japan, eg, 26% [(63 − 50 = 13 days)/50 days × 100] (*δ*′ = 1.5*δ*).

The effects of screening (eg, increases in life expectancies) were calculated for difference of *δ*′ (= 1.0*δ*–3.0*δ*) to test the sensitivity. There were considerable variations, but the screening strategy including women in their 40s remained optimal. Figure 3 shows the absolute effects compared with the relative effects of extended average life expectancy. Extended average life expectancy in the group aged 40–74 years (vs. 50–74 years) in *δ*′ = 1.5*δ* increased by 26% (13 days) and 22% (25 days) in Japan and the United States, respectively, under biennial screening conditions; however, the numbers of false-positive results increased 65% and 53% in Japan and the United States, respectively.

## DISCUSSION

### Comparison of annual vs. biennial screening

In Japan, biennial screening for breast cancer of women aged 40–49 years has been conducted since 2004. Our study indicates that biennial screening maintained 82% of the death reduction obtained by annual screening and reduced the number of false-positive mammographies by about half. Similarly, Mandelblatt et al concluded that biennial screening was the best procedure, as a biennial strategy maintains approximately 81% (range: 67%–99%) of the death reduction obtained with annual screening, while harmful effects—number of women with false-positive mammography results—are reduced by about half.^3^ Other modeling studies showed concordant results.^39^^,^^40^ Furthermore, a large-scale observational study reported that the risk for advanced breast cancer at the time of diagnosis in women aged 40 years increased only slightly in the biennial screening group compared with the annual screening group.^41^ Therefore, the findings in our study are consistent with previous studies, which support the screening interval recommended in Japan.

### Comparison of the range of ages for screening

The recommendation level (eg, recommendable, conditionally recommendable, or unrecommendable) of breast cancer screening for women aged 40–49 years must be considered separately in each country, particularly considering that the proportion of patients in their 40s is notably higher in Japan than in the United States. However, if we compare at absolute levels, incidences in both countries are nearly equal. Looking only at absolute incidence may justify applying the change in screening policy in the United States to Japanese women. However, the proportion of earlier stages in outpatient care is higher in Japan than in the United States. If these earlier-stage outpatients are likely to be in their 40s, the advantage of inviting women in their 40s may be reduced.

Two important points must be accounted for when considering the recommendation level. One is the weight attributed to harmful effects; if false-positive cases are considered to be exceedingly harmful, participation in screening is less recommendable. The other factor is performance of screening. It is notable that the number of screenings required to detect one case of breast cancer increases 36% and that to avert one death increases 52% if women in their 40s are included compared to initiating screening at age 50. More specific screening results in fewer false-positive cases, thus reducing the influence of their harmful effects. The final decision regarding optimum screening strategy must balance these two points. In Japan, the use of ultrasound has been proposed as a means of mass screening.^42^ If ultrasound technology successfully resolves the problem of harmful effects, the balance might favor more mass screening in the future. Overdiagnosis must also be taken into account when considering the harmful effects of screening. If individuals without mortal cancer are subjected to medical treatment due to detection by screening, they may experience harmful effects. The proportion of overdiagnosis is reported to be one third of diagnosed cases^43^ or 10%–20%,^44^ and therefore is not negligible in most countries. However, there are no data on the frequency of overdiagnosis in Japan, so further investigation is required.

Although age to terminate screening is determined in most countries, such a rule is not available in Japan. The 2009 update to the USPSTF guidelines considered that evidence regarding the efficiency of screening in women aged ≥75 years was insufficient, and the age range recommended to receive screening was amended to 50–74 years.^1^ One harm caused by cancer screening is overdiagnosis,^43^^–^^46^ which has a greater impact on the benefit-harm balance in elderly patients diagnosed with breast cancer because benefits relatively decrease among these patients.^47^ The present study also found age-related reductions in benefits of breast cancer screening in Japan. If we emphasize efficiency, the recommended age for terminating breast cancer screening is 69 years in Japan. Therefore, we consider that reexamining screening ages is an important challenge to address, not only for women aged 40–49 years but also for elderly women in Japan.

Our study has several limitations that warrant mention. First, mortality by age group as estimated with our simulation model is higher than the observed statistics indicate. For survival rates of United States patients with breast cancer, we used data on patients who were diagnosed from 1985–1990.^26^^,^^27^ However, the rates in 2006 were improved by prevention and treatment.^48^ Therefore, survival rates used in the model may not reflect the latest breast cancer death rates. Under-reporting of breast cancer death may also explain the differences. Second, observed statistics (eg, actual incidence and mortality rates) were used in this model. Caution is therefore required in using analysis results from the present study because a variety of factors (eg, birth patterns in the future) may influence the accuracy of these statistics.

### Conclusions

Women aged 40–49 years in Japan benefit from mass screening due to the high incidence of breast cancer in this age group. However, screening participants in their 40s may be harmed by the low specificity of mammography in this age group (ie, high proportion of false positives). Whether or not screening of women in their 40s in Japan is justifiable must be carefully determined based on the quantitative balance of benefits and harms.

## ONLINE ONLY MATERIAL

Abstract in Japanese.
