# Supplementary material for: An Analysis of Mass Screening Strategies Using a Mathematical Model: Comparison of Breast Cancer Screening in Japan and the United States
Source: J Epidemiol. 2015 Feb 5;25(2):162–71. doi: 10.2188/jea.JE20140047 (PMC4310878; doi:10.2188/jea.JE20140047)
Supplement: Abstract in Japanese. [file je-25-162-s001.pdf]

数理モデルによる集団検診条件の分析：日本と米国における乳がん検診の比較

恒松美輪子[1)]、梯正之[1)]
１）広島大学大学院 医歯薬保健学研究院 健康情報学研究室

【背景】
2009 年、米国予防医学専門委員会（USPSTF）が 40 歳代の定期的なマンモグラフィ検診に関して推奨グレードを下げたが、わが国では、2004 年から 40 歳代の乳がん検診の受診が推奨されている。本研究の目的は、両国の異なる背景を考慮すれば、最適な検診条件が異なるのかどうか、根拠を示して検討することである。

【方法】
乳がん自然史に基づいて、いくつかの段階を時間の経過とともに確率的に移行するように設定された数理モデルを使用して、様々な条件下（毎年と隔年の検診間隔、検診の開始年齢と終了年齢）での検診成績を評価した。利益としては、減少した死亡者数と延長された平均余命、不利益としては、偽陽性者数を計算した。

【結果】
隔年検診で、検診開始年齢を 50 歳代から 40 歳代にした場合、平均余命の延長効果は、日本では 13 日（26%）、米国では 25 日（22%）であった。一方、偽陽性者数は、日本では 65%、米国では 53%増加した。さらに、1 人の乳がんを発見するために必要な検診受診者数と 1 人の乳がん死亡を防ぐために必要な受診者数は、40 歳代を含めた時より 50 歳代以上に限定した方がより少なかった。わが国で 40 歳代を乳がん検診の対象年齢にするかどうかの正当性は、利益と不利益の評価を明確にしなければ決定できなかった。

【結論】
わが国で 40 歳代を検診の対象年齢にするかどうかは、利益と不利益の量的なバランスにかかっており、慎重に決定することが必要である。

キーワード：乳がん、集団検診、数理モデル、利益、不利益
